# A randomized controlled trial to test the effectiveness of two technology-enhanced diabetes prevention programs in primary care: The DiaBEAT-it study

**DOI:** 10.3389/fpubh.2023.1000162

**Published:** 2023-02-24

**Authors:** Fabio A. Almeida, Wen You, Fabiana A. Brito, Thais F. Alves, Cody Goessl, Sarah S. Wall, Richard W. Seidel, Brenda M. Davy, Mark H. Greenawald, Jennie L. Hill, Paul A. Estabrooks

**Affiliations:** ^1^Department of Health Promotion, University of Nebraska Medical Center, Omaha, NE, United States; ^2^Department of Public Health Sciences, University of Virginia, Charlottesville, VA, United States; ^3^Center for Clinical Epidemiology and Population Health, Marshfield Clinic Research Institute, Marshfield, WI, United States; ^4^Department of Human Nutrition, Foods and Exercise, Virginia Tech, Blacksburg, VA, United States; ^5^Department of Psychiatry, Carilion Clinic, Roanoke, VA, United States; ^6^Department of Family and Community Medicine, Carilion Clinic, Roanoke, VA, United States; ^7^Department of Populational Health Sciences, University of Utah, Salt Lake City, UT, United States; ^8^Department of Health and Kinesiology, University of Utah, Salt Lake City, UT, United States

**Keywords:** diabetes prevention, DVD, IVR, behavior change, weight loss, primary care

## Abstract

**Objective:**

To evaluate the effectiveness of two technology-enhanced interventions for diabetes prevention among adults at risk for developing diabetes in a primary care setting.

**Methods:**

The DiaBEAT-it study employed a hybrid 2-group preference (Choice) and 3-group randomized controlled (RCT) design. This paper presents weight related primary outcomes of the RCT arm. Patients from Southwest Virginia were identified through the Carilion Clinic electronic health records. Eligible participants (18 and older, BMI ≥ 25, no Type 2 Diabetes) were randomized to either Choice (*n* = 264) or RCT (*n* = 334). RCT individuals were further randomized to one of three groups: (1) a 2-h small group class to help patients develop a personal action plan to prevent diabetes (SC, *n* = 117); (2) a 2-h small group class plus automated telephone calls using an interactive voice response system (IVR) to help participants initiate weight loss through a healthful diet and regular physical activity (Class/IVR, *n* = 110); or (3) a DVD with same content as the class plus the same IVR calls over a period of 12 months (DVD/IVR, *n* = 107).

**Results:**

Of the 334 participants that were randomized, 232 (69%) had study measured weights at 6 months, 221 (66%) at 12 months, and 208 (62%) at 18 months. Class/IVR participants were less likely to complete weight measures than SC or DVD/IVR. Intention to treat analyses, controlling for gender, race, age and baseline BMI, showed that DVD/IVR and Class/IVR led to reductions in BMI at 6 (DVD/IVR −0.94, *p* < 0.001; Class/IVR −0.70, *p* < 0.01), 12 (DVD/IVR −0.88, *p* < 0.001; Class/IVR-0.82, *p* < 0.001) and 18 (DVD/IVR −0.78, *p* < 0.001; Class/IVR −0.58, *p* < 0.01) months. All three groups showed a significant number of participants losing at least 5% of their body weight at 12 months (DVD/IVR 26.87%; Class/IVR 21.62%; SC 16.85%). When comparing groups, DVD/IVR were significantly more likely to decrease BMI at 6 months (*p* < 0.05) and maintain the reduction at 18 months (*p* < 0.05) when compared to SC. There were no differences between the other groups.

**Conclusions:**

The DiaBEAT-it interventions show promise in responding to the need for scalable, effective methods to manage obesity and prevent diabetes in primary care settings that do not over burden primary care clinics and providers.

**Registration:**

https://clinicaltrials.gov/ct2/show/NCT02162901, identifier: NCT02162901.

## 1. Introduction

The Centers for Disease Control and Prevention (CDC) estimates that there are 34.2 million (10.5%) Americans with diabetes, in addition to the 88 million (34.5% of the population) with prediabetes in the United States, and strongly recommends healthcare approaches to prevent diabetes (1). Approximately 5–10% of individuals with prediabetes develop type 2 diabetes (T2D) yearly with an American Diabetes Association (ADA) (2) expert panel estimating that up to 70% of individuals with prediabetes will eventually progress to diabetes, further highlighting the importance of intervening (3). Finally, due to the continued growth of the obesity epidemic, the burden of prediabetes and diabetes are expected to continue to rise (4). As there is no known treatment available to cure diabetes and self-management for those with diabetes remains a challenge, the importance of prevention is paramount (5).

The Diabetes Prevention Program (DPP) was seminal in demonstrating that a modest weight loss achieved through diet and exercise was superior to medication in delaying the onset of T2D (6). The DPP program found that 30 min of physical activity per day 5 times a week coupled with a 5–10% weight loss resulted in a 58% reduction in the incidence of diabetes (6). Following on the success of the DPP, researchers have sought to determine the effectiveness of the DPP in more typical community and clinical settings (7–9). However, barriers to large-scale implementation of these adaptations still exist, where information on primary prevention and management of T2D is still limited (10). Recent studies (11, 12) have shown the lack of availability of these programs in underserved areas, with lifestyle coaches reporting lack of space, administrative support, sufficient allocation of their own time for the program, overall costs, and difficulty scheduling as barriers to broad dissemination of these programs (13). On the other hand, participants have reported distance, work schedules, lack of transportation and childcare needs as remaining issues that prevent them from fully engaging in these in-person group adaptations of the DPP (14–16).

To address these barriers, several interventions have used technology to successfully adapt and deliver the DPP. Several systematic reviews have shown that technology-based resources can optimize diabetes prevention intervention to achieve clinically significant weight loss (17). A review by Levine et al. (18) found that technology-assisted weight loss interventions that included some form of human coaching were successful in helping individuals lose weight in primary care settings. Another review (19) of technology-mediated diabetes prevention interventions found that these types of programs can result in a clinically significant amount of weight loss in patients with prediabetes. This review included studies that used a variety of technologies including DVDs, e-videos, web-based resources, videoconferencing, telephone calls, interactive voice response, text messages, e-counseling, email, and online group forums with a variety of Supplementary material (e.g., Physical Activity and Nutrition workbooks, log books, and in person group DPP). Joiner et al. (20) found similar results further supporting the effectiveness of technology-based interventions in helping individuals at risk for developing diabetes to lose clinically significant amounts of weight. However, questions remain regarding the effectiveness of eHealth interventions within primary care settings and in promoting weight loss maintenance or weight gain prevention (18, 21–23).

The original diaBEAT-it study (24) was a pragmatic clinical trial employing a hybrid preference/randomized control trial (RCT). The study focused primarily on the individual-level factors of reach, effectiveness, maintenance, and cost (24) but each active intervention was designed for broad dissemination and scalability within and across healthcare and, potentially, public health systems. The overarching goal of the study was to determine the effectiveness and maintenance of effects of two technology-enhanced interventions relative to standard care (SC) in reducing body weight within the context of a traditional RCT, while concurrently determining the relative reach of these two interventions within the context of a two-group preference design where participants had the option to choose which intervention they would like to participate in (Choice). The diaBEAT-it study has been fully described elsewhere (24). The purpose of the present paper is to evaluate the effectiveness of the two technology-enhanced interventions in supporting patients to reduce their body mass index (BMI) over an 18-month period relative to a minimal standard care intervention. We hypothesized that compared to minimal standard care, each intervention would result in greater mean reduction in BMI over 18 months.

## 2. Design and methods

Patients at risk for developing diabetes were randomly assigned (2-1) by the project manager to either an RCT or Choice study arm using a blocked (groups of 4) randomization table stratified by sex created by the study statistician. Patients in the RCT arm were further randomized (1-1-1) to one of three groups: (1) a 2-h small group class designed to help patients develop a personal action plan to prevent diabetes (SC) (25); (2) a 2-h small group class plus automated telephone calls using an interactive voice response system (IVR) to help participants initiate weight loss *via* the promotion of a healthful diet and regular physical activity, and maintain their behavior changes over a period of 12 months (Class/IVR); or (3) a DVD with same content as the class plus the same IVR calls over a period of 12 months (DVD/IVR).

This paper presents weight related outcomes associated with the randomized control trial arm of the DiaBEAT-it study (24). We powered our study to detect statistically significant body weight changes at 6 and 12 months in Class/IVR and DVD/IVR when compared with the SC group within the RCT design. Sample size was determined by using the average weight loss and standard deviations found in our previous studies (25–27) for the 6-month effect and averages from the literature (28, 29) for the 12-month effect. As such, assuming a correlation of 0.5 between repeated measures, we estimated that a sample size of 78 participants per group would give us a 90% power to detect a minimum detectable difference in change in weight of 2.3 lbs. at 6 months and 2.7 lbs. at 12 months. The goal for enrollment was 120 participants per group to achieve a sample size of 78 after an estimated 35% attrition at 18 months. The trial design and methods have been described in detail elsewhere (24). Supplementary Figure 1 provides the CONSORT information for the RCT study arm. This study and protocol were approved by the Carilion Clinic Institutional Review Board and was registered at clinicaltrials.gov (NCT02162901).

### 2.1. Participant eligibility and recruitment

Potential participants were initially identified through a Carilion Clinic electronic health records (EHR) query of primary care patients between January 2014 and August 2015 (24). EHR eligibility included patients that over the previous 12 months were 18 years of age and older, BMI ≥ 25, had ICD-9 codes for prediabetes, glucose intolerance, metabolic syndrome, and/or obesity while excluding those with ICD-9 codes indicating diagnosed diabetes, congestive heart failure, and coronary artery disease. A list of patients meeting initial eligibility criteria were sent to their physicians for final approval. All approved patients were recruited *via* a physician letter providing general information about the study and went through telephone screening for final eligibility determination. During the phone screening, a research assistant reiterated key points of the letter, answered any questions, and determined diabetes risk and study eligibility. Diabetes risk was determined using the Diabetes Risk Calculator (DRC) (30). Individuals with a score of 5 or higher are considered to be in particularly greater risk and were the target for recruitment.

Individuals were eligible if they were 18 years of age or older with a BMI of at least 25 kg/m^2^ (BMI > 22 for Asian), spoke English, were not pregnant or planning to become pregnant in the following 18 months, were not diagnosed with T2D, congestive heart failure, or coronary artery disease, had no contraindication for physical activity (PA) or weight loss, had access to a phone, and had a DRC test score indicative of high risk for developing T2D (Score of 5 or higher). Eligibility was broadly defined to allow for most typical primary care patients to be eligible to participate in the study.

Prior to the baseline visit, the project manager created sealed opaque envelopes with group assignment information according to the blocked (groups of 4) randomization table stratified by gender created by the study statistician to blind research staff to intervention assignment. Informed consent procedures were initiated during the screening telephone call with participants receiving the informed consent *via* mail prior to their initial visit so they could prepare for the first study visit. These procedures were completed at the time of the first study visit with participants (1) receiving information on the risks and benefits of participating on the trial, (2) being given the opportunity to ask questions, clarifications, and raise any concerns, and (3) being informed of the interventions of interest and their rights as a research subject. All assessments took place after full consent was given by study participants. Once all assessments were completed, a research assistant randomized participants in the RCT study arm to one of the three study groups using the previously created envelopes. Participants randomized to SC received information about the class and a workbook. Those randomized to the Class/IVR group received a workbook and were assisted by a research assistant in signing into an IVR account to select days and times best for their calls and setup a security PIN. Research assistants also helped participants in completing an initial test of the system to familiarize themselves with the IVR calls. Participants randomized to the DVD/IVR group received a workbook, a DVD and a brief instruction on how to use the TV to navigate the DVD in addition to the IVR system setup. Finally, all randomized participants received $25.00 as a thank you for their time in completing the baseline assessments.

### 2.2. Interventions

#### 2.2.1. Standard care

Participants in the SC comparison group took part in a 2-h small group session class (25) taught by two trained Carilion Clinic employees (Certified Diabetes Educators and Registered Dietitians). This class has been offered for the past 6 years and although they are available to all Carilion Clinic patients, for the purpose of the project, separate classes to each intervention group were offered. As such, both groups attended project specific classes for their given study group (SC or Class/IVR). The content, format, and individuals teaching these classes did not differ from the currently taught classes. Participants in the SC group received no additional intervention contact after the initial class. They were contacted 6, 12, and 18 months following their class date for follow-up assessments. During the class participants were encouraged to develop their own personal action plan to preventing T2D by setting a goal of losing 10% of their current weight over 12 months and to be physically active for 60 min, 5 days per week. The personal action plan also included a listing of motivational reasons to prevent diabetes, personal goals for weight management, physical activity, and healthful eating, identifying barriers, strategies to overcome barriers, and upholding accountability for these goals through a commitment to enlist friends and/or family members in the change process (25). Class instructors provided detailed information on current recommendations for physical activity and healthy eating (*MyPlate* guidelines) and gave a workbook covering all 22 session topics following a similar curriculum as developed by the original DPP. The class is fully described elsewhere (24).

#### 2.2.2. Class/IVR group

Participants in this group attended the 2-h class described above, received a workbook, completed a “Live” counseling call (31), and received 22 tailored IVR calls over a period of 12 months with the final 6 months focusing on maintenance and relapse prevention based on DPP's after Core program. This intervention was designed to help participants initiate moderate weight loss through physical activity and healthful eating and maintain these behavior changes. All participants developed a personal action plan with the goal of losing 10% of their current weight in 12 months and being physically active for 60 min a day, 5 days per week. Workbook content topics focused on achieving a balanced diet through the reduction of fat and caloric intake plus adding regular physical activity to enhance initial weight loss and prevent weight regain. Additionally, we used the 5 A's model to assist participants in setting physical activity and healthful eating goals necessary for weight loss and maintenance (32). One week after class completion, participants received a telephone call lasting 45–60 min to reinforce learning objectives and provide further clarifications (31). Research assistants delivered this call using teach-to-goal and teach-back strategies to allow participants to describe key intervention concepts (i.e., MyPlate guidelines, types, and length of physical activity) using their own words and provide additional rounds of education until the participant demonstrated a firm understanding of the information. For those participants that did not attend the initial 2-h class, the research assistants provided the full content of the class and assisted them in creating their personal action plan. One week after the live telephone call the participants began receiving IVR support calls. There were 22 IVR calls lasting between 15 and 30 min with 8 weekly calls, followed by 8 biweekly calls and 6 monthly calls focusing on maintenance and relapse prevention. Participants were required to complete one call before moving on to the next call, as such, it was not possible to skip IVR calls and content. For those participants that did not complete an IVR call, reminder contacts using telephone, text, and email were used for up to 2 weeks to try and get participants back on track. Each IVR call included an assessment of current weight, PA, and dietary behaviors, feedback on goal progression, content related to the session topic (i.e., Move Those Muscles, Being Active: A Way of Life, Healthy Eating With MyPlate, Be A Fat Detective), teach to goal reinforcement of key messages, and a homework assignment. New action plans were created every month (Calls 4, 8, 12, and 16) through Call 16 and then on every call during the maintenance and relapse prevention phase. This included updating goals, identifying new barriers, selecting strategies to resolve barriers, and goal setting-feedback loops.

#### 2.2.3. DVD/IVR group

This group was identical to the Class/IVR group but was initiated with a DVD that replicated the class content. The DVD included the following segments: (1) What is pre-diabetes? (2) What are the risk factors for diabetes? (3) Developing your DiaBEAT-it action plan, (4) Goal setting for physical activity and healthy eating, (5) putting together a toolbox of resources, and (6) making a commitment to change. The DVD was about 60 min in duration with several planned pauses to allow for completion of activities. This replicated the 5 A's approach that guided the class and included the completion of an action plan page in the accompanying workbook. Finally, the DVD included an appendix with additional free-of-charge, online nutrition and physical activity informational videos. Participants received their live counseling call within 7 days of being given the DVD. Similar to the Class/IVR group, those participants that reported not watching the DVD the research assistants provided full information and guided them through developing their personal action plan during the “Live” call. The IVR structure and content was the same as described above.

### 2.3. Outcome measures

Trained research assistants unaware of group assignment collected data at baseline, 6, 12, and 18 months. The primary outcome was change in BMI from baseline to 18 months. Secondary outcomes included percentage of participants achieving weight loss goals of 5% or more, changes in percent weight reduction as well as maintenance of those changes at 12 and 18 months. Height was assessed in stocking feet with a calibrated stadiometer with a fixed vertical backboard and adjustable headboard. Weight was assessed with the calibrated Health-O-Meter 2101KL digital stand-on scale (www.homscales.com). Body Mass Index was calculated in kg/m^2^. Demographic characteristics were collected using a computer-based questionnaire (https://surveymethods.com/). Research assistants were available on site to answer any questions and help participants with potential computer/survey issues. All assessments took place at a research facility.

### 2.4. Statistical analysis

Statistical analysis included descriptive statistics for age, sex, race, ethnicity, education, income, health literacy, employment, health insurance, Diabetes Risk Calculator (DRC), and weight status. Chi-square and independent *t*-tests were conducted to determine if any of the groups differed on baseline characteristics (Supplementary Table 1). Data were examined for the presence of outliers, violations of normality (for those continuous variables) and missing data. No violations of normality were detected. Between group differences in changes in BMI and other weight outcomes were prespecified using intention-to-treat (ITT) analysis. To simultaneously account for individual effects regardless of the condition, we employed a linear mixed effect model to a multi-treatment framework (33) for the treatment effect analysis (34). To be specific, two group dummies are in the model along with assessment time dummies and their interactions. This model allows us to control error non-independence of over time assessment within the same individual and heteroskedasticity caused by between individual heterogeneity, and a-priori-determined covariates that are influencing factors of outcome-specific production. The goal was to make more robust inferences about the treatment effect of main outcomes of interest: for example, the effect of Class/IVR and DVD/IVR in reducing BMI over 18 months when compared to SC group. For those participants with missing outcome measurements, we replaced the missing data with their baseline value following the Baseline Carried Forward approach.

Additionally, we conducted analysis based on participants completing at least 4 sessions (i.e., meeting NDPP threshold for recognition standards) (35), at least 6 months (i.e., core intervention effects), and the full 12 months (i.e., post-core effects). For the purposes of these analyses, class and “Live-Call” completion were calculated based on attendance, DVD was based on participant self-report, and IVR call completion was based on the voice files for the lesson of the week being played (24). Further, for the dichotomous outcome measures (i.e., achieve 5% weight loss goal), we treated those models as linear probability models in order to retain the straight-forward treatment effect interpretation of the results by applying generalized linear models in the analysis. Means and standard deviations for all primary and secondary outcomes at baseline, 6, 12, and 18 months are also presented. All statistical analyses were conducted in Stata v16 and the 5% significance level was used.

## 3. Results

### 3.1. Participant enrollment and characteristics

Supplementary Figure 1 presents participant enrollment and retention at 6, 12, and 18 months. A total of 3,115 were identified as potentially eligible to join the study. Of those, 1,712 (55%) were reached by phone with 689 completing screening questions and 427 scheduling an initial study visit to determine full eligibility. A total of 358 patients were eligible to participate in the study with 334 (93%) completing full baseline assessments and being randomized (SC = 117, Class/IVR = 110, DVD/IVR = 107). The mean age of participants was 52.3 (±12.1) years with a mean BMI of 37.2 (±7.3) kg/m^2^ (Supplementary Table 1). At baseline, 68.1% of participants were female, 76.8% were non-Hispanic white, 20.0% were Non-Hispanic black, 25.8% had high school or lower education, and 55.8% were employed full time (Supplementary Table 1). Intervention groups (Class/IVR and DVD/IVR) participants were less likely to be retired (*P* = 0.036), had higher average diabetes risk scores (*P* = 0.019) and higher average BMI (*P* = 0.004) when compared with SC participants (Supplementary Table 1). Of the 334 participants that were randomized, 232 (69%) had study measured weights at 6 months, 221 (66%) at 12 months, and 208 (62%) at 18 months. Class/IVR participants were less likely to complete weight measures than SC or DVD/IVR (Supplementary Figure 1).

### 3.2. Weight loss

Supplementary Table 2 reports estimated mean changes in BMI and weight over an 18-month period. A total of eight participants (SC = 1, Class/IVR = 6, DVD/IVR = 1) were eliminated from full analysis due to becoming pregnant during trial (Supplementary Figure 1). ITT results show that at month 6, the mean ± SE change in BMI from baseline in DVD/IVR was significant with −0.94 ± 0.21 (*P* = 0.022 vs. SC; *P* = 0.450 vs. Class/IVR), significant in Class/IVR with −0.70 ± 0.24 (*P* = 0.206 vs. SC), and non-significant in SC with −0.33 ± 0.17. At month 12, the mean ± SE change in BMI from baseline in DVD/IVR was significant with −0.88 ± 0.20 (*P* = 0.058 vs. SC; *P* = 0.853 vs. Class/IVR), significant in Class/IVR with −0.82 ± 0.25 (*P* = 0.141 vs. SC), and non-significant in SC with −0.36 ± 0.19. At month 18, the mean ± SE change in BMI from baseline in DVD/IVR was significant with −0.78 ± 0.22 (*P* = 0.030 vs. SC; *P* = 0.550 vs. Class/IVR), significant in Class/IVR with −0.58 ± 0.23 (*P* = 0.160 vs. SC), and non-significant in SC with −0.18 ± 0.17.

At month 6, mean percent weight loss ± SE change from baseline was significant in all three conditions (DVD/IVR: −2.77 ± 0.48; Class/IVR: −1.42 ± 0.46; SC: −1.40 ± 0.42) with DVD/IVR significantly losing more weight than Class/IVR (*P* = 0.046) and SC (*P* = 0.031) (Supplementary Table 2). At month 12, the mean percent weight loss ± SE change remained significant in all three conditions (DVD/IVR: −2.56 ± 0.50; Class/IVR: −1.80 ± 0.50; SC: −1.47 ± 0.44) with no between group differences (Supplementary Table 2). At month 18, the mean percent weight loss ± SE change remained significant in all three conditions (DVD/IVR: −2.18 ± 0.54; Class/IVR: −1.27 ± 0.48; SC: −1.11 ± 0.44) with no between group differences (Supplementary Table 2). Finally, results show positive time effects for DVD/IVR (6M: 25.84%, 12M: 26.87%, 18M: 20.69%), Class/IVR (6M: 18.59%, 12M: 21.62%, 18M: 18.59%), and SC (6M: 15.94%, 12M: 16.85%, 18M: 16.85%) participants achieving 5% weight loss across all three timepoints with no treatment effect found across groups (Supplementary Table 2).

### 3.3. Intervention participation rates: CDC recognition standards

On average participants in the DVD/IVR group completed 15.5 (±8.6) sessions compared to 14.1 (±8.3) for Class/IVR. Approximately, 86.3% of participants in the intervention groups (DVD/IVR: 86.6%; Class/IVR: 86%) met the CDC threshold of completing at least 4 sessions with 48.4% (DVD/IVR: 52.6%; Class/IVR: 44%) staying in the program for at least 6 months, and 29.5% (DVD/IVR: 37.1%; Class/IVR: 21.5%) completing every session during the 12-month program. Average percent weight loss at 12 months for those meeting the CDC threshold were 3.24% (DVD/IVR) and 2.74% (Class/IVR) with 35.74% (DVD/IVR) and 33.44% (Class/IVR) achieving a 5% weight loss.

#### 3.3.1. Adverse events

During the trial, 40 adverse events (AE) were reported; 6 were classified as serious adverse events (SAE). The majority were associated with immune system disorders (allergic reactions–21). Additional categories included cardiac disorders (1), musculoskeletal disorders (3), general disorders (1), infections (1), injury or procedure complications (3), neoplasms benign, malignant and unspecified (1), nervous systems disorders (1), respiratory disorders (1), and vascular disorders (2). Twenty-one AEs were determined to be related to the study and 3 had insufficient information to make a determination. The 21 related AEs were all associated with a skin irritation as result of the application of the accelerometer used in the study. One SAE also associated with the application of the accelerometer led to a severe reaction and hospitalization. Overall events were equally balanced between groups, with 13 in SC, 11 in Class/IVR, and 14 in DVD/IVR.

## 4. Discussion and conclusion

The randomized control trial arm of our study demonstrated that two technology-enhanced diabetes prevention programs both led to modest reductions in body weight over an 18-month period. Most importantly, the DVD/IVR group showed significant reductions in BMI when compared to the SC group confirming our original hypothesis. However, there were no significant differences between Class/IVR and SC groups. Participants in the DVD/IVR group lost a mean 2.79 kg over 12 months with 26.9% of participants losing 5% or more of initial body weight in ITT analyses. These numbers improve for both technology-enhanced groups as the number of sessions attended increased.

Our results support the findings of several recent reviews on technology mediated DPPs (19, 20), eHealth obesity interventions (23), weight loss interventions in primary care (18, 36), and self-help weight loss interventions (22). Joiner et al. (20) found an estimated mean percent weight loss from baseline to 15 months to be −3.98% across the 22 studies included in the review. This magnitude of effect varied from −3.32% for stand-alone eHealth interventions to −4.49% for interventions with behavioral support given by a counselor remotely to −4.65% for interventions with behavioral support given by a counselor in-person.

When investigating the effects of eHealth obesity interventions, Hutchesson et al. (23) found that eHealth interventions demonstrated significantly greater weight loss (kg) than control groups (−2.70), or minimal intervention comparisons (−1.40). This review of 84 studies also showed significant weight loss for web-based interventions that incorporated non-eHealth components (−3.70); mobile interventions (−2.40) and web-based interventions delivered only using eHealth technologies (−2.21). Levine et al. (18) found 12 interventions that achieved weight loss (range: 0.08 kg −5.4 kg) compared to controls, 5–45% of patients losing at least 5% of baseline weight with trial duration and attrition ranging from 3 to 36 months and 6–80%, respectively. On another review (36) of 15 RCTs focusing on weight loss in primary care settings, the authors showed pooled results from meta-analysis indicating a mean weight loss of −1.36 kg at 12 months, and −1.23 kg at 24 months. Hartmann-Boyce et al. (22) found similar results in their meta-analysis of 23 studies of self-help interventions for weight loss in overweight and obese adults. They found that intervention participants lost significantly more weight than controls at 6 months (−1.85 kg) with no significant effect at 12 months (−0.76). They also showed that programs using some form of interactivity appeared to be more effective than controls at 6 months (−0.94 kg).

Taken altogether, our results support existing evidence on the effectiveness of technology mediated DPPs (19, 20), eHealth interventions for weight loss (18, 23, 36), and weight loss interventions in primary care settings (18, 36). Our intent-to-treat weight loss magnitude across all three conditions at 6 (SC: −1.52, Class/IVR: −1.70, DVD/IVR: −3.04), 12 (SC: −1.56, Class/IVR: −2.04, DVD/IVR: −2.79), and 18 months (SC: −1.15, Class/IVR: −1.46, DVD/IVR: −2.55) were well within the range found in these reviews (−0.08 to −3.76). Most importantly, our study presents results at 18 months with significant reductions in BMI, which represents a significant addition to current literature on the weight loss maintenance effect of technology enhanced interventions delivered within a primary care setting (18, 23, 36). Additionally, our attrition rates (31–38%) and percent of individuals achieving at least 5% weight loss in all study groups across all timepoints (ITT: 15.94–26.87%) are well within the range found by Levine et al. (18) when investigating technology-assisted weight loss interventions in primary care.

Additionally, while not the original purpose of this study, results from this trial seem to indicate that both the DVD/IVR and the Class/IVR groups could meet CDC recognition standards (35) minus the “Live” health coaching requirement. Most importantly, our CDC threshold results indicate similar results to the latest NDPP evaluation (37). We found that on average participants completed 15.5 sessions in DVD/IVR and 14.1 in Class/IVR (NDPP: 14) with 86.3% of participants in the intervention groups (NDPP: 86.6%) meeting the CDC threshold of completing at least 4 sessions, 48.4% (NDPP: 48.3%) staying in the program for at least 6 months, and 29.5% (NDPP: 10.4%) attending at least the full 12-month program. Average weight lost at 12 months for DVD/IVR participants meeting the CDC threshold was 3.24% compared to 2.74% for Class/IVR (NDPP: 3.6%) with 35.74% of DVD/IVR participants and 33.44% of Class/IVR (NDPP: 35.5%) achieving 5% weight loss goal.

These are important findings when considering that several barriers to large scale implementation remain for technology enhanced DPPs (19, 20) and weight loss interventions in primary care settings (18, 23, 36). In fact, recent studies (11, 12) have shown that the NDPP as currently delivered and its technology-based options are not able to reach a large proportion of the American population. These studies have shown a lack of availability of these programs in underserved areas (11, 12) where primary care is overburdened and under resourced, and when these programs are offered, they fail to attract a large and representative sample of the target population (12). Further, the spotty access to internet services and reliance on data plans presents a barrier to engagement in traditional eHealth programs requiring Internet connection (38). The use of smart automated telephone calls to deliver DPP content shows promise in addressing these issues. The IVR system addresses barriers at multiple implementation levels. At the organizational, setting level the IVR system reduces the need for space, staff time, overall costs, and scheduling barriers. At the organizational, staff-level the IVR system reduces staff burden of delivering NDPP, and difficulty on scheduling participants, and allows staff to spend more time building relationships with participants to improve overall engagement. At the individual level, the IVR system addresses issues of distance, lack of transportation, work schedules, unreliable access to the Internet, and childcare needs. This is particularly important for primary care settings in underserved communities where there is a lack of resources, (i.e., medically underserved areas, space, competing demands, and expertise) and geographic segregation makes it difficult to deliver the NDPP or weight loss programs (13–16).

The present trial is not without limitations. First, study participants in the DVD/IVR group presented significantly higher BMI with a higher proportion being at Class III Obesity status at baseline. While we used randomization procedures, we did not stratify by BMI status. Nevertheless, we accounted for these initial differences by using baseline BMI values as control variable in our models. Second, we had an overall high attrition rate. As such, our results must be seen with caution as up to 38% of our participants did not complete follow-up assessments. These numbers were particularly higher among Class/IVR participants reaching 50% at 18 months. Nonetheless, we used Intent-to-treat analysis to include all participants with a baseline value in our models. When comparing with other studies, we also see similar attrition rates for weight loss programs in primary care (18). Future studies should continue to investigate factors influencing participant engagement and retention in weight loss interventions delivered in primary care settings. Finally, our trial lasted only 18 months and did not include glycemic control (e.g., HbA_1_) or event based (e.g., T2D incidence) outcomes. Thus, long-term effects of the three groups await further investigation.

In closing, our findings show that a technology-enhanced diabetes prevention program was effective in reducing BMI at 6 months and maintaining these results at 12 and 18 months in a group of primary care patients at risk for developing T2D. The DiaBEAT-it interventions respond to the need for scalable, effective methods to manage obesity and prevent diabetes in primary care settings that do not over burden primary care clinics and providers. Further, the CDC requires the inclusion of a lifestyle coach in any in-person or technology-based program as one of the standards for recognition in its National Diabetes Prevention Program (35). Consideration of expanded program criteria to include fully-automated systems and or the possibility of engaging clinical staff as engagement agents instead of lifestyle coaches, may reduce the burden placed on many resource strapped primary care clinics and improve the potential for adoption and sustainability of DPP adaptations. Effective automated technologies such as DiaBEAT-it represent one of these strategies with the potential to serving a large, representative and geographically distant population, while decreasing the need for organizational resources and reliance on Internet availability.

## Data availability statement

The raw data supporting the conclusions of this article will be made available by the authors, without undue reservation.

## Ethics statement

The studies involving human participants were reviewed and approved by Carilion Clinic Institutional Review Board. The patients/participants provided their written informed consent to participate in this study.

## Author contributions

FA, WY, RS, BD, MG, JH, and PE contributed to the conception of the protocol and study design. FA, WY, FB, TA, CG, and SW were involved with the data collection and analysis. All authors were involved in writing the paper and had final approval of the submitted manuscript.
