# Supplementary material for: A randomized controlled trial to test the effectiveness of two technology-enhanced diabetes prevention programs in primary care: The DiaBEAT-it study
Source: Front Public Health. 2023 Feb 24;11:1000162. doi: 10.3389/fpubh.2023.1000162 (PMC9998510; doi:10.3389/fpubh.2023.1000162)

Supplementary Material

1 Supplementary Tables

Supplementary Table 1. Participant baseline characteristics by group.

Variable	SC N=117	Class/IVR N=110	DVD/IVR N=107	Overall N=334	*P* value
**Demographic variables**					
Age, mean (SD), y	54.2 (12.1)	51.4 (11.8)	51.2 (12.1)	52.3 (12.1)	.110
Female, %	68.1	68.3	67.9	68.1	.999
**Race/ethnicity**					
Caucasian, %	83.0	71.0	76.5	76.8	.113
African American, %	17.0	24.0	21.4	20.0	.438
Another race, %	0.0	5.0	2.0	2.3	.049
Hispanic or Latino, %	2.7	3.0	1.0	2.3	.580
**Education level, %**					.726
0 to 11th Grade	0.92	3.0	5.0	2.9	
High School/GED	22.9	24.0	21.8	22.9	
Some College	38.5	34.0	36.6	36.5	
College graduate	36.7	39.0	36.6	37.4	
**Annual household income, %**					.939
Less than $15,000	8.6	4.3	8.0	7.1	
$15,000 to $24,999	8.6	7.5	10.0	8.7	
$25,000 to $34,999	6.7	10.8	9.0	8.7	
$35,000 to $49,999	17.1	20.4	17.0	18.1	
$50,000 to $74,999	26.7	23.7	22.0	24.2	
$75,000 to $99,000	16.2	21.5	17.0	18.1	
$100,000 or more	16.2	11.8	17.0	15.1	
**Health literacy status, %**					.247
Limited literacy	6.7	3.1	0.9	3.6	
Possibility Limited Literacy	18.3	17.4	17.0	17.5	
Adequate literacy	75.0	79.6	82.1	78.9	
**Employment Status, %**					.036
Full-time	50.5	60.0	57.4	55.8	
Part-time	8.3	9.0	3.0	6.8	

Self-employed	6.4	2.0	10.9	6.5	
Out of work > 1 year	3.7	0.0	0.0	1.3	
Out of work < 1 year	0.9	3.0	1.0	1.6	
Homemaker	4.6	7.0	5.0	5.5	
Student	0.9	1.0	1.0	1.0	
Retired	22.0	14.0	12.9	16.5	
Disability	2.8	4.0	8.9	5.2	
**Insurance Status, %**					.082
Through employer	52.7	46.0	53.5	50.8	
Other's employer	10.0	23.0	16.8	16.4	
Purchased Plan	10.9	9.0	5.0	8.4	
Medicaid	7.3	13.0	5.9	8.7	
Military, CHAMPUS, VA	2.7	0.0	5.0	2.6	
Other Source	12.7	7.0	10.9	10.3	
No Coverage	3.6	1.1	3.0	2.6	
Don't know	0.0	1.1	0.0	0.3	
**Diabetes Risk Calculator, mean (SD)**	5.1 (1.5)	5.5 (1.4)	5.6 (1.6)	5.4 (1.5)	.019
**Weight, kg**	100.3 (19.3)	105.0 (20.4)	108.9 (26.5)	104.6 (22.4)	.016
**BMI, kg/m2**	35.5 (5.9)	37.5 (7.2)	38.8 (8.4)	37.2 (7.3)	0.004
**BMI Status, %**					.012
Overweight	12.1	9.6	11.3	11.0	
Class I Obesity	48.3	32.7	30.2	37.4	
Class II Obesity	17.2	31.7	20.8	23.0	
Class III Obesity	22.4	26.0	37.7	28.5	

a. CHAMPUS - Civilian Health and Medical Program of the Uniformed Services, VA – Veterans Affairs
b. BMI status: 25.0 – 29.9 = Overweight, 30.0 and above = Obesity.
c. SC – Standard Care
d. DVD – Digital Video Disc
e. IVR – Interactive Voice Response
f. Health Literacy Status: based on the Newest Vital Sign (NVS) health literacy assessment

Class I Obesity: BMI of 30 to < 35, Class II: BMI of 35 to < 40. Class III: BMI of 40 or higher.

Supplementary Table 2. Estimated mean ± SE change in weight related outcomes over an 18-month period in the intention-to-treat population.

		Mean ± SE			*P* Value	
Outcome Variable	SC N=116	Class/IVR N=104	DVD/IVR N=106	Class/IVR vs Class	DVD/IVR vs Class	DVD/IVR vs Class/IVR

Intent-to-treat						
**BMI, kg/m2**						
Baseline	37.09 (0.04)	37.07 (0.04)	37.13 (0.04)	-	-	-
At 6 months	36.76 (0.17)	36.37 (0.23)	36.19 (0.20)	.168	.025*	.570
At 12 months	36.73 (0.18)	36.25 (0.25)	36.25 (0.20)	.111	.073	.998
At 18 months	36.90 (0.17)	36.48 (0.23)	36.35 (0.22)	.128	.039*	.681
**Change in BMI**						
At 6 months	-0.33 (0.17)	-0.70** (0.24)	-0.94*** (0.21)	.206	.022*	.450
At 12 months	-0.36 (0.19)	-0.82*** (0.25)	-0.88*** (0.20)	.141	.058	.853
At 18 months	-0.18 (0.17)	-0.58** (0.23)	-0.78*** (0.22)	.160	.030*	.550
**Weight change, kg**						
At 6 months	-1.52*** (0.42)	-1.70** (0.55)	-3.04*** (0.61)	.801	.041*	.102
At 12 months	-1.56*** (0.46)	-2.04*** (0.60)	-2.79*** (0.60)	.523	.102	.379
At 18 months	-1.15** (0.44)	-1.46** (0.55)	-2.55*** (0.63)	.653	.067	.191
**Weight change, %**						
At 6 months	-1.40*** (0.42)	-1.42** (0.46)	-2.77*** (0.48)	.975	.031*	.046*
At 12 months	-1.47*** (0.44)	-1.80*** (0.50)	-2.56*** (0.50)	.615	.101	.294
At 18 months	-1.11* (0.44)	-1.27** (0.48)	-2.18*** (0.54)	.800	.123	.212
**Achieve at least 5% Weight Loss, %**						
At 6 months	15.94 (3.52)	18.59 (3.89)	25.84 (4.36)	.615	.078	.215
At 12 months	16.85 (3.61)	21.62 (4.20)	26.87 (4.39)	.391	.080	.386
At 18 months	16.85 (3.62)	18.59 (3.97)	20.69 (4.13)	.746	.484	.715

a. * p<0.05 ** p<0.01 *** p<0.001
b. SC – Standard Care

c. DVD – Digital Video Disc
d. IVR – Interactive Voice Response

1.1 Supplementary Figures

Supplementary Figure 1. DiaBEAT-it Trial: Consort diagram of procedures for the randomized controlled trial arm.

Figure 1. DiaBEAT-it Trial: Consort diagram of procedures for the randomized controlled trial arm

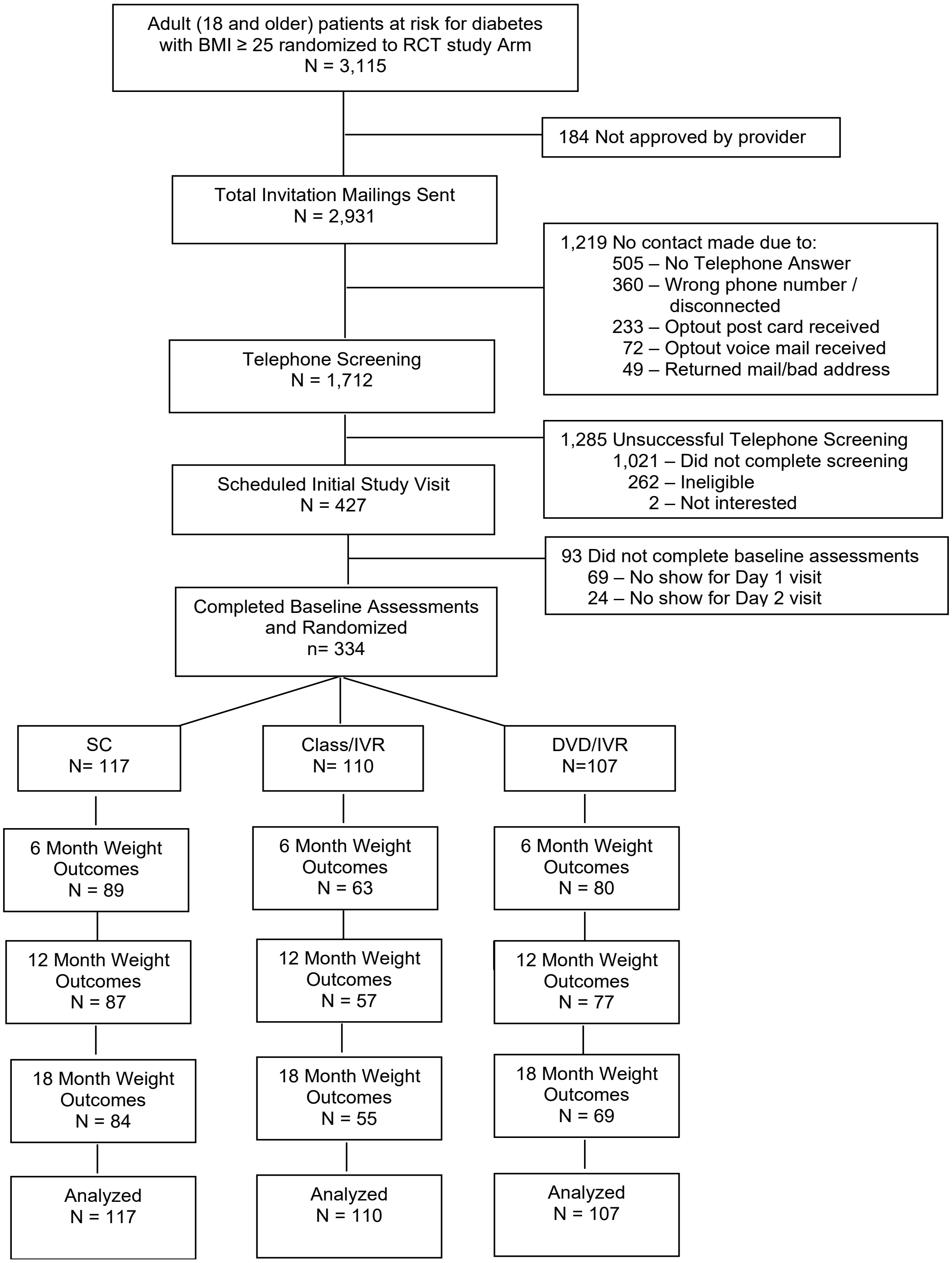

a. BMI - Body Mass Index
b. DVD - Digital Video Disc
c. IVR - Interactive Voice Response

Supplementary Figure 2. Changes in BMI over an 18-month period.

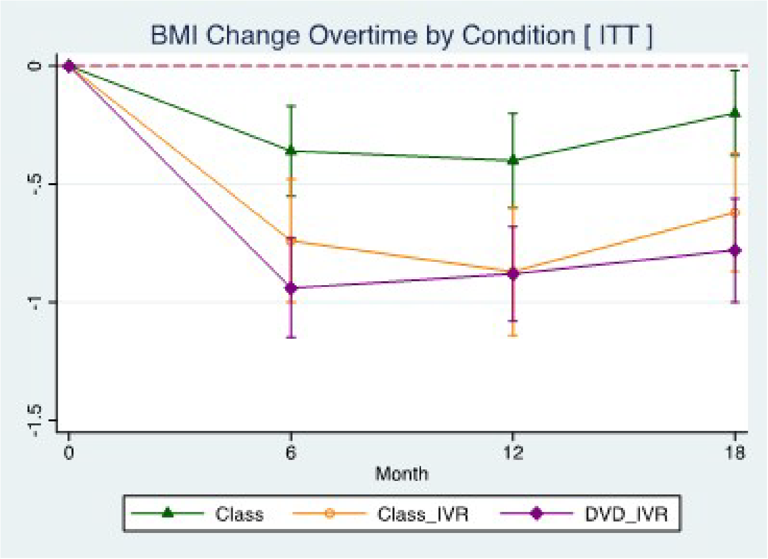

Supplement: Supplementary file 1 [file Data_Sheet_1.PDF]
